# Methicillin-Susceptible *Staphylococcus aureus* ST398, New York and New Jersey, USA

**DOI:** 10.3201/eid1804.111419

**Published:** 2012-04

**Authors:** José R. Mediavilla, Liang Chen, Anne-Catrin Uhlemann, Blake M. Hanson, Marnie Rosenthal, Kathryn Stanak, Brian Koll, Bettina C. Fries, Donna Armellino, Mary Ellen Schilling, Don Weiss, Tara C. Smith, Franklin D. Lowy, Barry N. Kreiswirth

**Affiliations:** University of Medicine and Dentistry of New Jersey, Newark, New Jersey, USA (J.R. Mediavilla, L. Chen, B.N. Kreiswirth);; Columbia University, New York, New York, USA (A.-C. Uhlemann, F.D. Lowy);; University of Iowa, Iowa City, Iowa, USA (B.M. Hanson, T.C. Smith);; Jersey Shore University Medical Center, Neptune, New Jersey, USA (M. Rosenthal);; Beth Israel Medical Center, New York (K. Stanak, B. Koll);; Albert Einstein College of Medicine, Bronx, New York, USA (B.C. Fries);; North Shore University Hospital, Manhasset, New York, USA (D. Armellino, M.E. Schilling);; New York City Department of Health and Mental Hygiene, New York (D. Weiss)

**Keywords:** Staphylococcus aureus, MSSA, ST398, CC398, livestock-associated, human, infection, bacteria, New York, New Jersey, staphylococci, United States

**To the Editor:** Clinical infections with livestock-associated *Staphylococcus aureus* sequence type (ST) 398 have been reported in Europe, Canada, and the People’s Republic of China (*1*), as well as the Caribbean (*2*,*3*), and Colombia (*4*). Most reports describe infection with methicillin-resistant *S. aureus*; relatively few describe infection with methicillin-susceptible *S. aureus* (MSSA). In the United States, colonization of healthy adults by ST398 has been reported in Iowa (*5*) and in New York, New York (*2*); MSSA infections have been anecdotally reported in St. Louis, Missouri (*6*), and The Bronx, New York (*7*). We describe 8 infections with MSSA ST398 in the New York City area during a 7-year period (2004–2010). Five infections with a related ST (ST291) from clonal complex (CC) 398 also were identified. These findings highlight the emergence of clinical infections with 2 distinct CC398 sequence types in the New York City area.

Retrospective typing of 4,167 clinical *S. aureus* isolates from various studies involving inpatients and outpatients in the New York City area identified 13 *mecA*-negative isolates with CC398-associated *spa* types (Table). Nine isolates were obtained from cultures of outpatients with skin and soft tissue infections; samples were submitted by physicians in the community. One isolate was associated with recurring skin and soft tissue infections in multiple body sites (BK21466); another was associated with genital infection (BK21732). Of the 4 ST398 isolates derived from bloodstream infections in hospitalized patients, 3 were recovered from intravenous drug users, 1 of whom died 1 day after admission for variceal bleeding (BK26722). Unlike the multidrug-resistant ST398 MSSA recently described in Colombia (*4*), most isolates in this study were susceptible to all antimicrobial drugs tested except penicillin, although several strains exhibited resistance to clindamycin and erythromycin. One isolate (BK23527) was submitted as oxacillin resistant (MIC ≥4 μg/mL) but lacked the *mecA* gene, which suggested that another mechanism was contributing to the resistance phenotype.

**Table:** Characteristics of *Staphylococcus aureus* clonal complex 398 isolates, New York and New Jersey, USA, 2004–2010*

Isolate†	Year	Geographic location‡	Submitting institution§	Isolate source	Antimicrobial resistance	PFGE pattern	*spa* type	*spa* repeats	Ridom type	ST
BK13684	2004	Monmouth County, NJ	Laboratory A	Wound	PEN	–	539	XKAOAOBQO	t034	398
BK18505	2006	Manhattan	Laboratory A	Wound	–	A1	109	XKAOAOBO	t571	398
BK21466	2007	Staten Island	Laboratory A	Arm, face, leg, buttocks	–	A2	109	XKAOAOBO	t571	398
BK21732	2007	Manhattan	Laboratory A	Genital	PEN, CLI, ERY	A3	109	XKAOAOBO	t571	398
BK27037	2007	The Bronx	Hospital A	Blood, lung abscess	PEN, CLI, ERY	A4	109	XKAOAOBO	t571	398
BK23527	2008	Manhattan	Hospital B	Blood, buttocks	PEN, ERY, OXA	–	109	XKAOAOBO	t571	398
BK26722	2009	Manhattan	Hospital B	Blood	CLI, ERY	A1	109	XKAOAOBO	t571	398
BK31274	2010	Nassau County, NY	Hospital C	Blood, sternum	PEN, CLI, ERY	A1	1376	XKAOBO	t1451	398
BK13771	2004	Somerset County, NJ	Laboratory A	Wound	PEN, ERY	B4	716	XKBBM	t2993	291
BK13451	2004	Union County, NJ	Laboratory A	Wound	PEN	B2	718	XKBQBMM	t1149	291
BK19382	2006	Staten Island	Laboratory A	Right ear	PEN	B1	865	XKBQBBM	t2313	291
BK21746	2007	Manhattan	Laboratory A	Torso	PEN, ERY	B1	208	XKBQBBMM	t937	291
BK22183	2007	Manhattan	Laboratory A	Axilla	PEN	B3	208	XKBQBBMM	t937	291

*Antimicrobial drug susceptibilities were obtained from submitting institutions (unavailable for BK18505 and BK21466). PFGE was performed by using *Cfr*9I, with patterns assigned on the basis of 80% similarity cutoffs (BioNumerics version 6.5, Applied Maths, Austin, TX, USA); BK13684 and BK23527 were unavailable for PFGE analysis. *spa* typing was performed by using eGenomics software (www.egenomics.com), and Ridom *spa* types were assigned by using the SpaServer Web site (www.spaserver.ridom.de). Multilocus sequence typing was performed as described (http://saureus.mlst.net). PFGE, pulsed-field gel electrophoresis; *spa*, staphylococcal protein A; ST, sequence type; wound, skin and soft-tissue infections from unspecified body sites; PEN, penicillin; CLI, clindamycin; ERY, erythromycin; OXA, oxacillin (resistance >4 μg/mL was reported for BK23527, but *mecA* was not detected by real-time PCR). †BK27037 has been described (*7*). ‡Manhattan, Staten Island, and the Bronx are boroughs of New York, New York. §Laboratory A is a large outpatient commercial laboratory serving the metropolitan New York, New York, area.

Multilocus sequence typing confirmed 8 isolates as ST398 (3–35–19–2–20–26–39); 5 isolates were assigned to ST291 (3–37–19–2–20–26–32), a double-locus variant of ST398 (Figure A1, panel A). Most of the ST398 strains were *spa* type 109 (t571), described in MSSA carriage isolates from New York City (*2*) and MSSA infections from China (*1*), France (*8*), Martinique (*3*), the Dominican Republic (*2*,*3*), and Colombia (*4*). BURP (based upon repeat pattern) analysis clustered all of the *spa* types into *spa*-CC t571 (Figure A1, panel B); ST398 isolates clustered with *spa* type 109 (t571), whereas ST291 isolates clustered with *spa* type 865 (t2313). Pulsed-field gel electrophoresis was also performed on the 11 available isolates. Although the ST291 isolates were sensitive to digestion with *Sma*I, pulsed-field gel electrophoresis was performed with *Cfr*9I to compare all isolates simultaneously. As expected, the ST398 and ST291 isolates clustered separately (data not shown); 4 distinct patterns were observed within each cluster (Table). Only the ST398 isolates were positive for a CC398 lineage-specific PCR that targets the unique restriction-modification system *sau1*-*hsdS1* (*9*), further highlighting the differences between ST291 and ST398. None of the isolates harbored the genes coding for Panton-Valentine leukocidin.

Because of the retrospective nature of the findings, epidemiologic information for each isolate was limited. One patient (BK19382) reported travel to the Dominican Republic; Caribbean nationality was reported for BK27037 (Puerto Rico) and BK31274 (Trinidad). The cases described here occurred in urban and suburban settings, reflecting the likelihood that exposure to livestock was relatively low; however, travel history was unknown for most of the patients. Previous reports have linked ST398 transmission to other reservoirs, including companion animals, live animal food markets, and commercial meat products (*1*,*2*). However, data from a recent genome sequencing study suggest that MSSA ST398 is human in origin (*10*); other evidence suggests that certain lineages, particularly *spa* type 109 (t571), might circulate at low levels in humans in the absence of livestock exposure (*8*).

Our findings seem to support the hypothesis of low-level ST398 MSSA prevalence, and further surveillance might uncover additional cases of colonization or infection with ST398- and ST291-related strains in the New York City area. For example, active surveillance cultures performed at one of the 3 hospitals during January–March 2009 detected 7 additional ST398 and 3 additional ST291 isolates among 260 MSSA carriage strains (data not shown). In addition to the intrinsic virulence exhibited by ST398 MSSA in previous studies, the potential to acquire resistance to multiple classes of antimicrobial drugs (*1*,*4*,*10*), as well as virulence factors such as Panton-Valentine leukocidin (*8*), warrants continued surveillance in light of recent ST398 methicillin-resistant *S. aureus* outbreaks in health care settings (*1*).

## Figures and Tables

**Figure A1:**
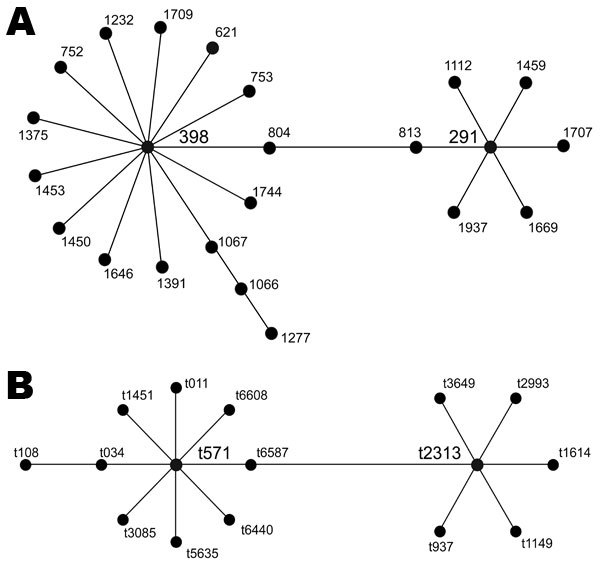
A) Schematic representation of *Staphylococcus aureus* clonal complex (CC) 398, generated by eBURST version 3 from the *S. aureus* multilocus sequence typing database (http://saureus.mlst.net) as of September 2011. Subgroup founders sequence type (ST) 398 (3–35–19–2–20–26–39) and ST291 (3–37–19–2–20–26–32) are double locus variants, despite the depiction of intervening STs ST804 (3–35–19–2–20–26–2) and ST813 (3–37–19–2–20–26–2). Subgroup founders are denoted in larger font. B) *spa* clonal complex (*spa*-CC) t571, generated by using the integrated BURP (based upon repeat patterns) algorithm in Ridom StaphType software, version 1.5 (www.ridom.de/staphtype). ST398-associated *spa* types (t034, t571, t1451) and ST291-associated *spa* types (t937, t1149, t2313, t2993) described in the article cluster separately as in panel A. Subgroup founders are denoted in larger font.
